# RUNX2 Phosphorylation by Tyrosine Kinase ABL Promotes Breast Cancer Invasion

**DOI:** 10.3389/fonc.2021.665273

**Published:** 2021-05-31

**Authors:** Fang He, Yoshinori Matsumoto, Yosuke Asano, Yuriko Yamamura, Takayuki Katsuyama, Jose La Rose, Nahoko Tomonobu, Ni Luh Gede Yoni Komalasari, Masakiyo Sakaguchi, Robert Rottapel, Jun Wada

**Affiliations:** ^1^Department of Nephrology, Rheumatology, Endocrinology and Metabolism, Okayama University Graduate School of Medicine, Dentistry and Pharmaceutical Sciences, Okayama, Japan; ^2^Princess Margaret Cancer Center, University Health Network, University of Toronto, Toronto, ON, Canada; ^3^Department of Cell Biology, Okayama University Graduate School of Medicine, Dentistry, and Pharmaceutical Sciences, Okayama, Japan

**Keywords:** ABL - Abelson murine leukemia viral oncogene homolog, Runx2 (runt-related transcription factor 2), tyrosine, phosphorylation, invasion

## Abstract

Activity of transcription factors is normally regulated through interaction with other transcription factors, chromatin remodeling proteins and transcriptional co-activators. In distinction to these well-established transcriptional controls of gene expression, we have uncovered a unique activation model of transcription factors between tyrosine kinase ABL and RUNX2, an osteoblastic master transcription factor, for cancer invasion. We show that ABL directly binds to, phosphorylates, and activates RUNX2 through its SH2 domain in a kinase activity-dependent manner and that the complex formation of these proteins is required for expression of its target gene MMP13. Additionally, we show that the RUNX2 transcriptional activity is dependent on the number of its tyrosine residues that are phosphorylated by ABL. In addition to regulation of RUNX2 activity, we show that ABL transcriptionally enhances RUNX2 expression through activation of the bone morphogenetic protein (BMP)-SMAD pathway. Lastly, we show that ABL expression in highly metastatic breast cancer MDA-MB231 cells is associated with their invasive capacity and that ABL-mediated invasion is abolished by depletion of endogenous RUNX2 or MMP13. Our genetic and biochemical evidence obtained in this study contributes to a mechanistic insight linking ABL-mediated phosphorylation and activation of RUNX2 to induction of MMP13, which underlies a fundamental invasive capacity in cancer and is different from the previously described model of transcriptional activation.

## Introduction

Tyrosine kinase signaling networks are required for multiple cellular functions including growth, survival and angiogenesis during tumorigenesis (1). It has been shown by the Cancer Genome Atlas (TCGA) and other studies that the ABL kinase (Abelson murine leukemia viral oncogene homolog 1) is amplified and/or overexpressed in various invasive solid tumors including breast, lung, colon, and kidney carcinoma as well as melanoma (1–3), though the role of ABL in oncogenic activity remains to be determined.

Metastasis is a multistep process by which tumor cells disseminate from a primary tumor to distant secondary organs. During the process of metastasis, tumor cells interact with the extracellular matrix (ECM), produce matrix metalloproteinases (MMPs), degrade the ECM and displace the normal tissue with the expanded tumors as a consequence of invasion (4–11). Expression of MMPs in cancer cells is strongly associated with their invasive capacity, leading to poor prognosis (12–14). However, regulation of MMPs by transcription factors during metastasis has yet to be elucidated.

RUNX2, also known as core-binding factor 1 (Cbfa1), has been revealed to be a master transcription factor required for osteoblast differentiation since studies showed that mice lacking RUNX2 fail to undergo bone ossification due to defective osteoblastogenesis (15, 16). In our previous study, we showed that ABL potentiates the assembly and activation of the critical transcriptional complex of RUNX2 and TAZ (transcriptional co-activator with PDZ-binding motif) and drives osteocalcin expression and development of the osteoblast lineage (17). Activity of transcription factors is thus normally regulated through interaction with other transcription factors, chromatin remodeling proteins and transcriptional co-activators in a variety of distinct physiologic states (18–23).

In distinction to these well-established transcriptional controls of gene expression, we have uncovered a unique activation model of transcription factors between tyrosine kinase ABL and RUNX2 required for cancer invasion. We found that ABL directly binds to, phosphorylates, and activates RUNX2 through its SH2 domain in a kinase activity-dependent manner. We also found that the complex formation of these proteins is required for expression of its target gene MMP13. Additionally, we found that the RUNX2 transcriptional activity is dependent on the number of its tyrosine residues that are phosphorylated by ABL. In addition to regulation of RUNX2 activity, we found that ABL transcriptionally enhances RUNX2 expression through activation of the bone morphogenetic protein (BMP)-SMAD pathway. Lastly, we found that ABL expression in highly metastatic breast cancer MDA-MB231 cells is associated with their invasive capacity and that ABL-mediated invasion is abolished by depletion of endogenous RUNX2 or MMP13.

These findings contribute to a mechanistic insight linking ABL-mediated phosphorylation and activation of RUNX2 to induction of MMP13, which underlies a fundamental invasive capacity in cancer and is different from the previously described model of transcriptional activation.

## Materials and Methods

### Mice

We purchased BALB/c-nu/nu female mice from Charles River Laboratories. All of the mice were housed in groups of 3-5 per cage and maintained at 22°C under a 12:12 h light/dark cycle with free access to water and standard laboratory food (MF diet, Oriental Yeast Co., Tokyo, Japan). Animal experiments were conducted in accordance with institutional and NIH guidelines for the humane use of animals.

### Cell Cultures

All cultures were maintained in a 5% CO_2_ environment at 37°C. HEK 293T cells (ATCC) were cultured in DMEM (GIBCO) supplemented with 10% fetal bovine serum (FBS) (Sigma). MDA-MB231 cells (ATCC) were cultured in α-MEM (Nacalai Tesque) supplemented with 10% FBS. MDA-MB231 cells stably expressing luciferase were cultured in DMEM/F12 (Gibco) supplemented with 10% FBS. Saos-2 cells (ATCC) were cultured in McCoy’s 5A Modified Medium (GIBCO) supplemented with 15% FBS.

### Invasion Assay With Matrigel

Cell invasion was assayed using the Boyden chamber method with filter inserts (pore size, 8 µm) pre-coated with Matrigel in 24-well plates (BD Biosciences, Franklin Lakes, NJ) as described previously (24). Cells (8 × 10^4^ cells/insert) were seeded with α-MEM containing 0.5% FBS on the top chamber, and the bottom chamber was filled with α-MEM containing 10% FBS. After incubation for 24 h, cells that passed through the filter were fixed and stained by H&E staining. Invading cells were quantified by cell counting in five non-overlapping fields at ×10 magnification and presented as the average from three independent experiments.

### *In Vivo* Metastasis Assays

For *in vivo* imaging, MDA-MB231 cells stably expressing luciferase were infected with an shGFP- or sh*ABL*-expressing vector, and 1 × 10^6^ cells were injected into the lateral tail veins of BALB/c-nu/nu female mice. After 4 weeks, the presence of metastases was detected using the IVIS Imaging System (Xenogen, Alameda, CA) following intraperitoneal luciferin injection (150 mg/kg). Regions of interest from displayed images were identified and quantified as total photon counts or photons/s using Living Image^®^ software 4.0 (Xenogen).

### Histology

Lung tissues from mice were fixed in 10% neutral formalin, embedded in paraffin, sectioned, and stained with H&E.

### Reagents and Antibodies

Unless stated otherwise, all chemicals were purchased from Sigma. Antibodies were obtained from the following sources: anti-pABL (Y245) (Cell Signaling Technology), anti-ABL (BD Pharmingen), anti-Flag M2 (Sigma), anti-Actin (Santa Cruz Biotechnologies), anti-RUNX2 (MBL International) and anti-pTyr (4G10) (EMD Millipore) antibodies. Halt™ Protease and Phosphatase Inhibitor Cocktail was from Thermo Fisher Scientific.

### Plasmids

ABL (WT, PP or KD), TAZ and RUNX2 (WT or YF) plasmids were constructed as described previously (17). RUNX2 (add back) plasmids were generated by overlap extension PCR using primers with the desired mutations and cloning into the XbaI site of pEF Bos.

### RNA Extraction and Quantitative Real-Time PCR (qPCR) Analysis

Total cellular RNA was extracted using an RNeasy Plus Mini Kit (QIAGEN). A High-Capacity cDNA Reverse Transcription kit (Applied Biosystems) was used for reverse transcription, and qPCR was performed on a Step One Plus Real-Time PCR System (Applied Biosystems) using TaqMan Gene Expression assays (Applied Biosystems) for *Gapdh* (Hs02786624_g1), *MMP2* (Hs01548727_m1), *MMP9* (Hs00957562_m1) and *MMP13* (Hs00942584_m1). The relative expression of each mRNA was calculated by the ΔCt method.

### Expression of an FKBP-ABL Retroviral Vector

An FKBP-ABL retroviral vector was constructed as described previously (25). HEK293T cells were co-transfected with an empty vector control (Mock) or pMx-FKBP-ABL with pSV and pVSVG using the CalPhos Mammalian Transfection Kit (Clontech). Saos-2 cells were infected as described previously (25).

### Lentiviral Transduction

pLKO.1 lentiviral vectors expressing short hairpin RNAs (shRNAs) targeting *RUNX2* (sh*RUNX2*), *ABL* (sh*ABL*), *MMP13* (sh*MMP13*) or a nonspecific GFP sequence (shGFP) were co-transfected into HEK293T cells with pPAX2 and pVSVG (Addgene) using X-tremeGENE 9 transfection reagent (Roche). The virus was collected 48 hours after transfection, and cells were infected as described previously (25).

### Western Blot Analysis and Co-Immunoprecipitation

Cells were lysed with Nonidet P-40 (NP-40) buffer (20 mM Tris [pH 8.0], 137 mM NaCl, 1% NP-40, 2 mM EDTA) or RIPA buffer (50 mM Tris [pH 7.5], 150 mM NaCl, 1% NP40, 0.1% SDS, 0.25% sodium deoxycholate, 1 mM EDTA) supplemented with protease and phosphatase inhibitors. Lysates were cleared by centrifugation for 10 minutes at 14,000 rpm and 4°C. Immunoprecipitation was performed at 4 °C with the indicated antibodies, and the products were collected on Dynabeads^®^ Protein A or G (Life Technologies) as described previously (26, 27). For Western blotting, proteins in whole cell lysates (WCL) were resolved by SDS-PAGE and transferred to PVDF membranes (Immobilon; Millipore). The membranes were blocked in 5% BSA or 5% nonfat dried milk in PBST (PBS + 0.1% Tween-20). The images presented are representative of three independent experiments. The relative integrated density of each protein band was digitized by NIH image J.

### Transient Transfection

HEK293T cells were transiently co-transfected with RUNX2 plasmid with or without TAZ and ABL constructs using LipoD293™ DNA *In Vitro* Transfection Reagent (SignaGen Laboratories).

### Statistics

All results are shown as means ± SEM of data from at least three separate experiments. The data were subjected to ANOVA with Tukey–Kramer’s *post hoc* test or unpaired t-test with JMP^®^ 7 (SAS Institute Inc, USA) to determine differences. P values < 0.05 were accepted as statistically significant.

### Study Approval

All animal studies were approved by the Animal Research Council at Okayama University, Okayama, Japan.

## Results

### ABL Kinase Activity Is Required for RUNX2-Mediated MMP13 Expression

**Figure 1:**
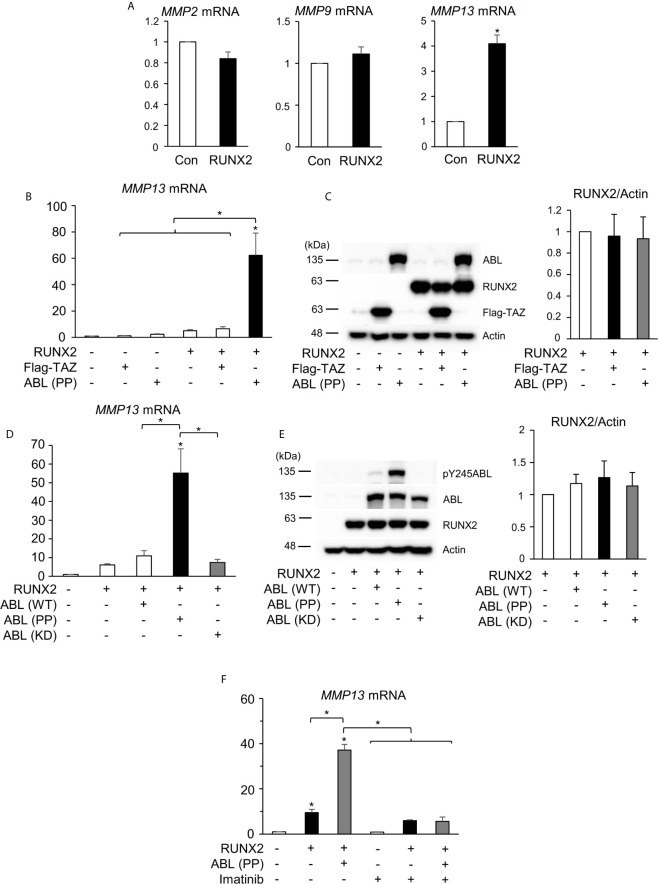
ABL kinase activity is required for RUNX2-mediated MMP13 expression. **(A)** Quantitative PCR analysis of *MMP2, 9, and 13* mRNA expression in HEK293T cells transfected with RUNX2. n = 3. **(B)** Quantitative PCR analysis of *MMP13* mRNA expression in HEK293T cells co-transfected with RUNX2 with or without TAZ or ABL (PP). n = 3. **(C)** HEK293T cells were co-transfected with RUNX2 with or without TAZ or ABL (PP). Whole cell lysates were probed with the indicated antibodies for Western blot analysis. **(D)** Quantitative PCR analysis of *MMP13* mRNA expression in HEK293T cells co-transfected with RUNX2 with or without ABL (WT, PP or KD). n = 3. **(E)** HEK293T cells were co-transfected with RUNX2 with or without ABL (WT, PP or KD). Whole cell lysates were probed with the indicated antibodies for Western blot analysis. **(F)** Quantitative PCR analysis of *MMP13* mRNA expression in HEK293T cells co-transfected with RUNX2 with or without ABL (PP) and cultured in the presence or absence of 10 μM imatinib for 24 hours. n = 3. P values were determined by the unpaired t-test **(A)** or ANOVA with Tukey–Kramer’s *post hoc* test **(B–F)**. Data are presented as means ± SEM. *P < 0.05.

Several MMPs have been reported to be transcriptionally regulated by RUNX2 in different physiologic states including tumorigenesis and bone metabolism (18, 28–30). We previously reported that ABL forms the RUNX2-TAZ transcriptional complex that is required for osteocalcin expression and osteoblast differentiation (17) and we hypothesized that RUNX2-mediated expression of MMPs lies downstream of the same regulatory system composed of TAZ and ABL observed in osteoblasts. We first confirmed that RUNX2 enhanced mRNA expression of MMP13 but not that of MMP2 or 9 in a 293T cell overexpression system (**Figures 1A**, **S1A**). However, in contrast to osteocalcin, co-expression of RUNX2 with the constitutively active form of ABL [ABL (PP)], but not TAZ, enhanced the expression level of MMP13 by tenfold (**Figures 1B**, **S1B**). The protein expression levels of RUNX2 were similar in the presence or absence of ABL (PP) (**Figure 1C**), indicating that the enhancing effect of ABL on RUNX2-mediated MMP13 expression was through elevation of RUNX2 transcriptional activity. Additionally, the kinase dead version of ABL [ABL (KD)] did not show this effect (**Figures 1D, E** and **S1C**). Lastly, we observed that the ABL kinase inhibitor imatinib rescued the level of RUNX2-mediated MMP13 expression activated by ABL (PP) to normal levels (**Figures 1F**, **S1D**). These findings demonstrate that ABL kinase activity, but not TAZ, is required for RUNX2-mediated MMP13 expression that is different from the control of osteocalcin expression by RUNX2.

### ABL Binds to, Phosphorylates, and Activates RUNX2 Through Its SH2 Domain

**Figure 2:**
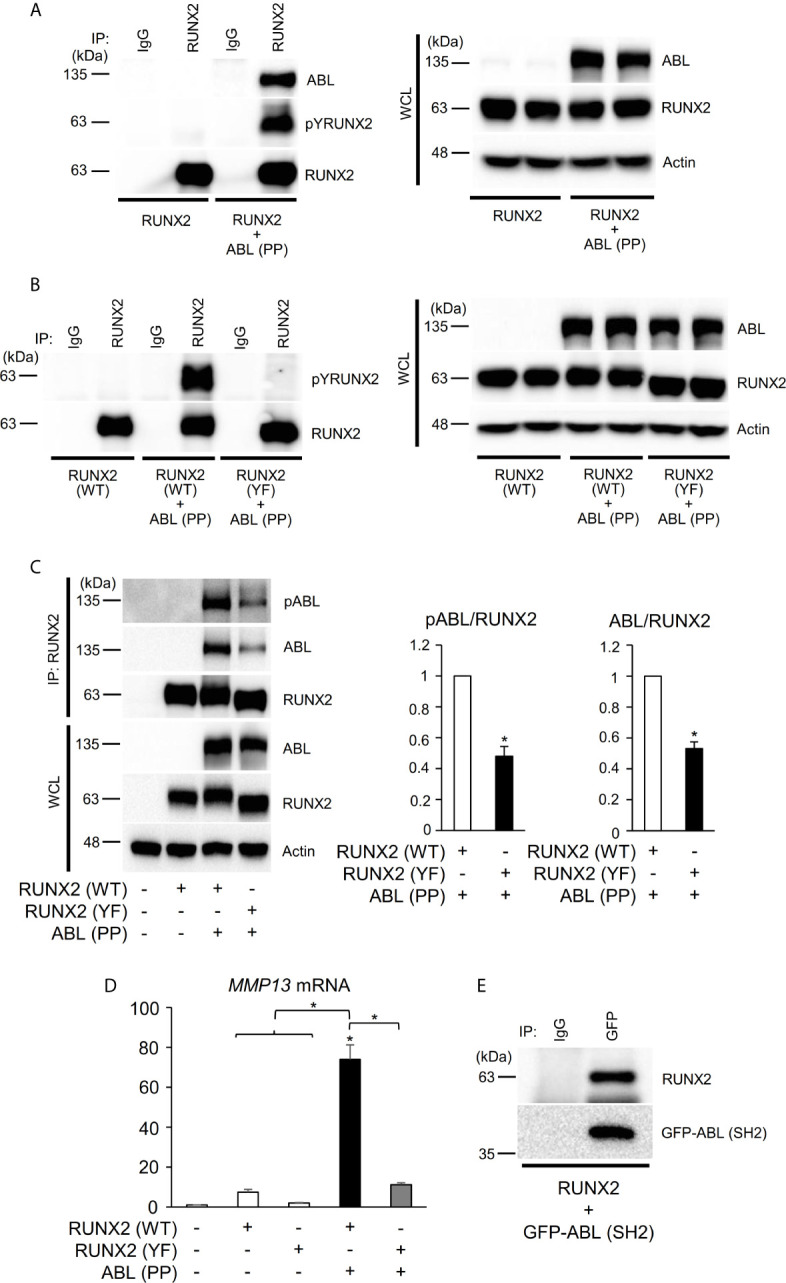
ABL binds to, phosphorylates, and activates RUNX2 through its SH2 domain. **(A–C)** HEK293T cells were co-transfected with wild-type (WT) or all tyrosine to phenylalanine mutant (YF) RUNX2 with or without ABL (PP). RUNX2 immune complexes were probed with an anti-phosphotyrosine (4G10), anti-pY245ABL, anti-ABL or anti-RUNX2 antibody. Whole cell lysates (WCL) were probed with the indicated antibodies for Western blot analysis. **(D)** Quantitative PCR analysis of *MMP13* mRNA expression in HEK293T cells co-transfected with RUNX2 (WT or YF) with or without ABL (PP). n = 3. **(E)** HEK293T cells were co-transfected with RUNX2 with or without GFP-ABL (SH2). GFP-ABL (SH2) immune complexes were probed with an anti-RUNX2 or anti-GFP antibody. P values were determined by ANOVA with Tukey–Kramer’s *post hoc* test. Data are presented as means ± SEM. *P < 0.05.

We next investigated the mechanism by which ABL regulates RUNX2-mediated MMP13 expression. We previously found that ABL interacted with and phosphorylated RUNX2, which was required for osteocalcin expression in osteoblasts (17). Consistent with this finding, ABL (PP) formed a complex with and tyrosine-phosphorylated wild-type RUNX2 [RUNX2 (WT)] but not the all tyrosine to phenylalanine mutant RUNX2 (YF) in the 293T overexpression system (**Figures 2A, B**). Interestingly, we observed that the RUNX2 (YF) mutant poorly formed a complex with ABL compared to RUNX2 (WT) and was transcriptionally inactive (**Figures 2C, D**, **S2A**), suggesting that a tyrosine residue(s) of RUNX2 is required for formation of the ABL-RUNX2 complex. To confirm this possibility, we generated a truncated form of the ABL-SH2 domain that binds to phosphorylated tyrosines of its substrate and observed the complex formation of RUNX2 and the SH2 domain (**Figure 2E**). These findings demonstrate that ABL controls the RUNX2 transcriptional activity for MMP13 expression through direct interaction and its tyrosine phosphorylation.

### RUNX2 Transcriptional Activity Is Dependent on the Number of Its Tyrosine Residues Phosphorylated by ABL

**Figure 3:**
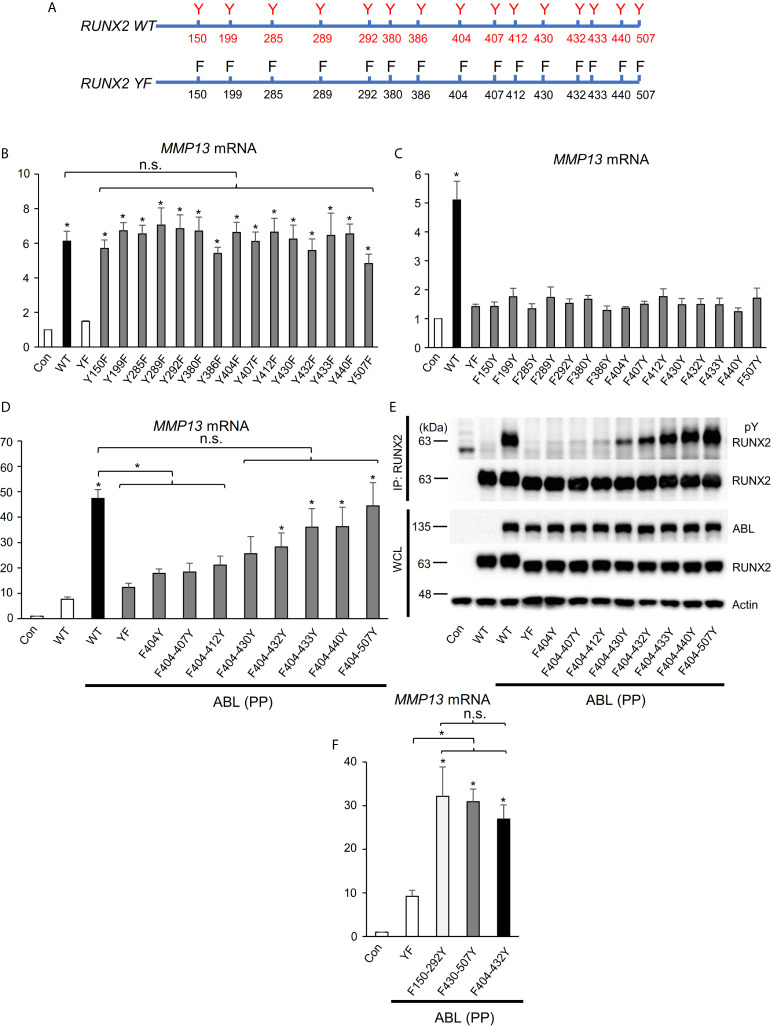
RUNX2 transcriptional activity is dependent on the number of its tyrosine residues phosphorylated by ABL. **(A)** Schematic models of RUNX2 (WT) and RUNX2 (YF). **(B–D, F)** Quantitative PCR analysis of *MMP13* mRNA expression in HEK293T cells co-transfected with the indicated constructs. n = 3. **(E)** HEK293T cells were co-transfected with the indicated constructs and RUNX2 immune complexes were probed with an anti-4G10 or anti-RUNX2 antibody. P values were determined by ANOVA with Tukey–Kramer’s *post hoc* test. Data are presented as means ± SEM. *P < 0.05. ns, no significance.

We next investigated the molecular mechanism by which ABL activates RUNX2 through its phosphorylation. RUNX2 contains fifteen tyrosines (Y150-507) (**Figure 3A**), and we first created one tyrosine to phenylalanine mutant variants or one tyrosine add-back variants to the RUNX2 (YF) mutant (**Figure 3A**) to determine which tyrosine or tyrosines are sufficient to mediate RUNX2 activation. Neither one tyrosine mutant variants nor one tyrosine add-back variants showed reduction (**Figures 3B**, **S3A**) or restoration (**Figures 3C**, **S3B**) of RUNX2-mediated MMP13 expression compared to RUNX2 (WT), suggesting that more than one tyrosine is required for RUNX2 transcriptional activity. We therefore created a variant in which half of the fifteen tyrosines were added back to the RUNX2 (YF) mutant (F404-507Y). To identify minimum tyrosines required for RUNX2-mediated MMP13 expression, we created several tyrosine add-backs to the RUNX2 (YF) mutant and found that not only the RUNX2 (F404-507Y) mutant but also five tyrosines add-back RUNX2 (Y404-432) were sufficient to restore RUNX2 activation and phosphorylation mediated by ABL (**Figures 3D, E** and **S3C**). Lastly, we queried whether the RUNX2 transcriptional activity is dependent on the number of its tyrosine residues that are phosphorylated by ABL. We created two variants in which five of the fifteen tyrosines were added back to the RUNX2 (YF) mutant [F150-292Y (far left) and F430-507Y (far right)] and observed that the transcriptional activities for MMP13 expression of these variants as well as RUNX2 (F404-432Y) were similarly increased compared to that of RUNX2 (YF) (**Figures 3F**, **S3D**). These findings suggest that the number of tyrosine residues phosphorylated by ABL may be important for its transcriptional activity for MMP13.

### ABL Regulates RUNX2 Expression Through Control of the BMP-SMAD Pathway

**Figure 4:**
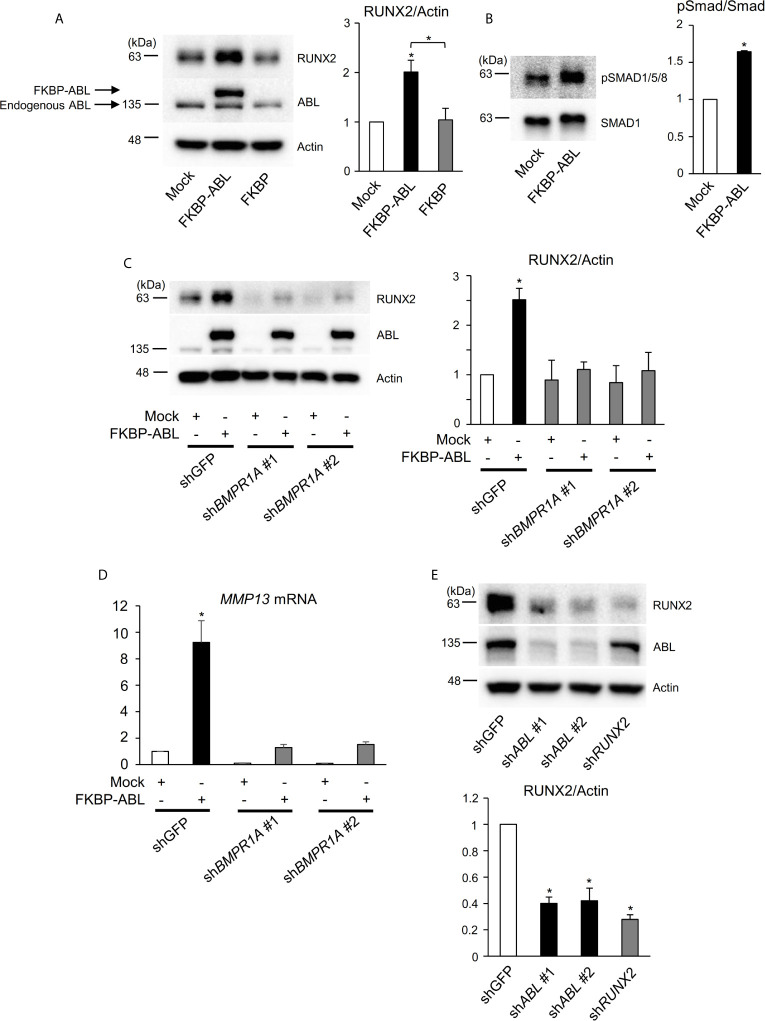
ABL regulates RUNX2 expression through control of the BMP-SMAD pathway. **(A, B)** Saos-2 cells were infected with an empty vector control or an FKBP-ABL- or FKBP-expressing retroviral vector. Whole cell lysates were probed with the indicated antibodies for Western blot analysis. **(C)** Saos-2 cells were infected with an empty vector control or an FKBP-ABL-expressing retroviral vector in the presence of shGFP or sh*BMPR1A*. Whole cell lysates were probed with the indicated antibodies for Western blot analysis. **(D)** Quantitative PCR analysis of *MMP13* mRNA expression in cells in **(C)**. n = 3. **(E)** MDA-MB231 cells were infected with an shGFP-, sh*ABL-* or sh*RUNX2*-expressing vector. Whole cell lysates were probed with the indicated antibodies for Western blot analysis. P values were determined by ANOVA with Tukey–Kramer’s *post hoc* test. Data are presented as means ± SEM. *P < 0.05.

Interestingly, we found in the present study that the ABL kinase enhances not only RUNX2 transcriptional activity but also its protein expression. We used an FKBP chimeric form of ABL for which activity is enhanced by the small molecule FK1012 (17) and we observed that FKBP-ABL but not Mock or FKBP potentiated the expression of RUNX2 in a human osteosarcoma cell line, Saos-2 (**Figure 4A**). We queried whether active ABL accelerated the RUNX2 protein expression level through activation of the BMP-SMAD signaling pathway that transcriptionally targeted RUNX2 and we observed that SMAD1/5/8 was activated in Saos-2 cells expressing FKBP-ABL (**Figure 4B**). Additionally, the increased levels of RUNX2 protein as well as MMP13 transcripts in FKBP-ABL-expressing Saos-2 cells were abolished in cells in which endogenous BMP receptor type IA (BMPR1A) was depleted (**Figures 4C, D** and **Figure S4A**). Lastly, we observed that the RUNX2 protein expression level was reduced in MDA-MB231 cells in which endogenous ABL was depleted (**Figure 4E**). These findings demonstrate that ABL enhances RUNX2 expression through activation of the BMP-SMAD signaling pathway, forms a complex with RUNX2, and accelerates its transcriptional activity through tyrosine phosphorylation that is required for MMP13 expression.

### ABL-Mediated RUNX2 Expression and Phosphorylation Regulate Breast Cancer Invasion

High expression levels of MMPs are associated with the capacity of invasion and metastasis in various cancer cells (12–14). In the present study, we showed that expression and tyrosine phosphorylation of RUNX2 mediated by ABL regulate MMP13 expression. Previous studies showing that MMP13 is required for invasion and metastasis of breast cancer cells prompted us to query whether the ABL-RUNX2 transcriptional complex potentiated breast cancer invasion. Consistent with our results in 293T cells, depletion of ABL or RUNX2 reduced MMP13 expression in MDA-MB231 cells (**Figure S5A**). To determine whether the ABL-RUNX2 complex controls metastasis, we performed an *in vitro* invasion assay and observed that depletion of these proteins inhibited the ability of invasion (**Figure S5B**)

We next queried whether the ABL-RUNX2 complex controlled metastasis to distant organs in mice. The lung was the first organ to which intravenously injected breast cancer cells metastasized due to being trapped by pulmonary capillary vessels (31). As shown in **Figures S5C, D**, we observed that mice injected with ABL-depleted MDA-MB231 cells had a smaller number of lung metastases than those in mice injected with control cells, suggesting that ABL-mediated RUNX2 expression and activity regulate the invasive capacity as well as seeding and growth of breast cancer cells in lung metastasis.

### Invasive Activity Accelerated by ABL Is Rescued in RUNX2- or MMP13-Depleted Breast Cancer Cells

**Figure 5:**
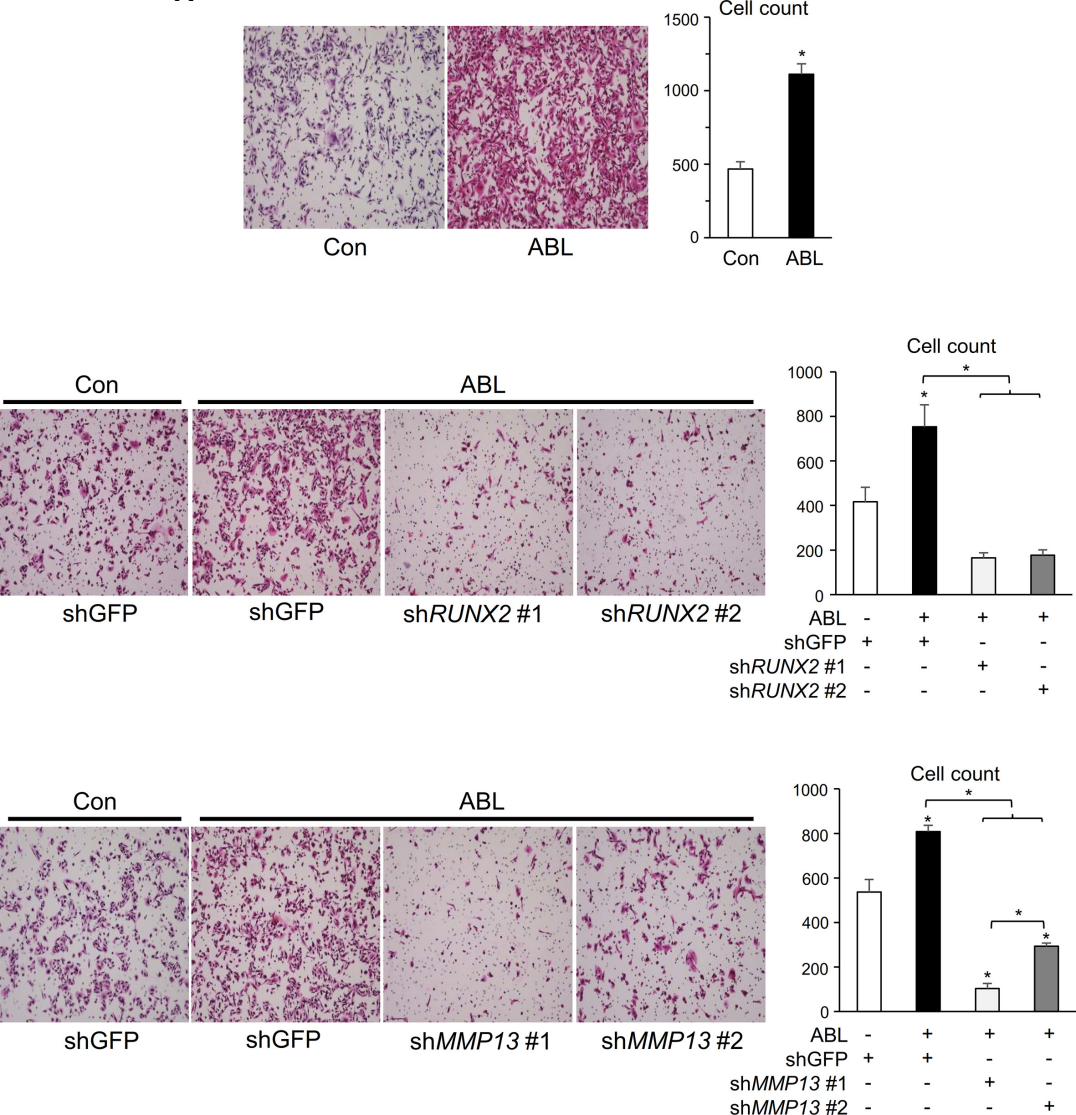
Invasive activity accelerated by ABL is rescued in RUNX2- or MMP13-depleted breast cancer cells. **(A)** MDA-MB231 cells co-transfected with or without ABL were subjected to a Matrigel invasion assay, and invading cells in five independent regions were counted. Representative photographs were taken at 10 × magnification. **(B)** MDA-MB231 cells co-transfected with or without ABL were infected with an shGFP- or sh*RUNX2*-expressing vector and subjected to a Matrigel invasion assay. Invading cells in five independent regions were counted. Representative photographs were taken at 10 × magnification. **(C)** MDA-MB231 cells co-transfected with or without ABL were infected with an shGFP- or sh*MMP13*-expressing vector and subjected to a Matrigel invasion assay. Invading cells in five independent wound regions were counted. Representative photographs were taken at 10 × magnification. P values were determined by the unpaired t-test **(A)** or ANOVA with Tukey–Kramer’s *post hoc* test (B,C). Data are presented as means ± SEM. *P < 0.05.

We finally determined whether the ability of active ABL to enhance invasive capacity was contingent on RUNX2 and MMP13. In contrast to the results of ABL depletion shown in **Figure S5B**, overexpression of ABL enhanced the ability of invasion in MDA-MB231 cells compared to control cells (**Figures 5A**, **S6A**). On the other hand, knockdown of RUNX2 or MMP13 abolished the invasive ability enhanced by ABL in MDA-MB231 cells (**Figures 5B, C** and **S6B, C**). These findings conclusively demonstrate that ABL controls RUNX2 expression and activation through its tyrosine phosphorylation, which is required for MMP13 expression and the invasive program.

## Discussion

### Tyrosine Phosphorylation of RUNX2 by ABL Is Required for Its Transcriptional Activity and Invasive Capacity in Breast Cancer

It is well established that RUNX2 activity is controlled by various factors including other transcription factors and transcriptional co-activators. The hippo pathway component TAZ and RUNX2 form a transcriptional complex, which drives development of the osteoblast lineage, while TAZ coordinately represses PPARγ-dependent gene transcription that is important for adipocyte lineage commitment (32). In our previous study, we showed that ABL potentiates RUNX-TAZ complex formation that is required for osteocalcin expression and osteoblast differentiation (17). ABL and TAZ are reciprocally stabilized through exclusion of their respective E3-ubiquitin ligases, SMURF1 and β-TrCP (17). Stabilized ABL phosphorylates TAZ and enhances its interaction with RUNX2 and TEAD1, leading to osteoblast differentiation and expansion, respectively (17). On the other hand, the TAZ paralog YAP has been reported to be phosphorylated by SRC, leading to suppression of RUNX2 activity (33). Thus, RUNX2 activity, which is generally controlled by transcriptional co-activators, regulates cellular identity in mesenchymal origin cells.

In distinction to these regulatory mechanisms of the transcription factors, we have uncovered a previously undescribed model showing that ABL, but not TAZ or other factors, directly binds to, phosphorylates, and activates RUNX2 through its SH2 domain in a kinase activity-dependent manner. ABL-RUNX2 complex formation is required for expression of its target gene MMP13 and subsequent invasive capacity in metastatic breast cancer cells. Additionally, we found the RUNX2 transcriptional activity is dependent on the number of its tyrosine residues that are phosphorylated by ABL. Although the PY motif (PPxY) in RUNX2 (Y412) is critical for interaction with the WW domain-containing proteins TAZ and YAP (34, 35), neither one tyrosine mutant variants (Y412F in RUNX2 WT) nor one tyrosine add-back variants (F412Y in RUNX2 YF) affected RUNX2-mediated MMP13 expression in our study (**Figures 3B, C**), indicating that phosphorylation of several tyrosines in RUNX2 by ABL is linked to its transcriptional activity through different mechanisms. It was shown in previous studies that multiple tyrosine phosphorylation of the BIK1 tyrosine kinase controls its kinase activity (36) and that tyrosine phosphorylation of the cytoplasmic domain of CD79a/b changes its helical propensity and structure (37). The results of our study and those previous studies suggest that the RUNX2 transcriptional activity is dependent on the number of phosphorylated tyrosine residues that could change its formation and interaction with the transcriptional coactivator and/or the target genes.

This study has provided evidence showing that tyrosine phosphorylation is directly involved in activation of the transcription factor and has provided an insight linking the ABL-RUNX2 transcriptional complex to the regulation of invasive capacity during metastasis. Further studies will be required to examine the roles of the phospho-switch for activation of RUNX2.

### ABL Controls RUNX2 Expression Through Regulation of the BMP-SMAD Pathway

In addition to control of RUNX2 transcriptional activity, ABL transcriptionally increases RUNX2 expression through activation of the BMP-SMAD pathway. Overexpression of ABL increased phosphorylation of SMAD1/5/8 and subsequent RUNX2 expression, while depletion of BMPR1A abolished this effect, leading to suppression of MMP13 expression. It has been reported that activation of the non-canonical BMP-ERK pathway leads to p16^INK4a^ upregulation and cell senescence in *ABL^-/-^* mesenchymal osteoprogenitor cells (38). Our findings provide a new mechanistic insight into the role of ABL for the BMP-SMAD pathway in cancer cells and expand the concept that BMPs and their target genes lie downstream of the tyrosine kinase ABL in multiple lineages.

### ABL-RUNX2-MMP13 Axis in Cancer and Other Physiologic States

In the present study, we uncovered a new regulatory mechanism of cancer invasion by linking ABL to RUNX2 and MMP13. We showed that RUNX2-mediated MMP13 expression lies downstream of ABL and that depletion of ABL in breast cancer cells inhibits invasive ability. Additionally, invasive capacity accelerated by ABL was abolished by depletion of RUNX2 or MMP13, demonstrating that the regulation of invasion and metastasis by ABL is at least in part through the control of RUNX2 and MMP13 expression. It has been reported that ABL phosphorylates proliferating cell nuclear antigen (PCNA), a component of DNA replication and maintenance, and controls tumorigenesis (39). It has also been reported that ABL kinases protected tumor cells from apoptosis induced by TNF-related apoptosis-inducing ligand (TRAIL) (40) and that a high expression level of RUNX2 is associated with poor prognosis in patients with osteosarcoma (41). Interestingly, ABL-mediated phosphorylation of RUNX1, another member of the Runt-related transcription factor family, inhibited RUNX1-mediated megakaryocyte maturation through the control of its transcriptional activity (42). The results of those previous studies and the present study suggest that the ABL-RUNX2-MMP13 axis is involved in the metastatic program in some patients with breast cancer expressing ABL and/or RUNX2 and that the use of ABL-specific inhibitors may be a new therapeutic strategy in those patients.

In addition to the oncogenic effects of these proteins, RUNX2 has been shown to be associated with cartilage degradation in patients with osteoarthritis and with osteoclast recruitment in bone remodeling (29, 30). Furthermore, MMP13 is known to be associated with tissue destruction in rheumatoid arthritis (43). The present study suggests that RUNX2-mediated MMP13 expression controlled by ABL may lie downstream of not only cancer biology but also other physiologic pathways. Further studies will be required to investigate the roles of the ABL-RUNX2-MMP13 axis for the maintenance of homeostasis.

We have reported a unique mechanistic strategy whereby the interplay between ABL and RUNX2 activates MMP13 expression needed to “lock-in” invasion and metastasis.

## Data Availability Statement

The raw data supporting the conclusions of this article will be made available by the authors, without undue reservation.

## Ethics Statement

The animal study was reviewed and approved by The Animal Research Council at Okayama University.

## Author Contributions

FH and YM designed the experiments. FH performed experiments and analyzed the results. YA, YY, TK, JLR, NT, NLGYK, MS, RR and JW performed specific experiments and analyzed the results. FH and YM wrote the manuscript. All authors contributed to the article and approved the submitted version.

## Funding

This work was supported by a grant from the Japan Society for the Promotion of Science, the Princess Takamatsu Cancer Research Fund, the Kobayashi Foundation, the Okinaka Memorial Institute for Medical Research and the Ryobi Teien Memory Foundation. FH is supported by a scholarship to overseas students funded by the Uehara Memorial Foundation.

## Conflict of Interest

The authors declare that the research was conducted in the absence of any commercial or financial relationships that could be construed as a potential conflict of interest.
