# Corrigendum: RUNX2 Phosphorylation by Tyrosine Kinase ABL Promotes Breast Cancer Invasion

**DOI:** 10.3389/fonc.2021.729192

**Published:** 2021-07-20

**Authors:** Fang He, Yoshinori Matsumoto, Yosuke Asano, Yuriko Yamamura, Takayuki Katsuyama, Jose La Rose, Nahoko Tomonobu, Ni Luh Gede Yoni Komalasari, Masakiyo Sakaguchi, Robert Rottapel, Jun Wada

**Affiliations:** ^1^ Department of Nephrology, Rheumatology, Endocrinology and Metabolism, Okayama University Graduate School of Medicine, Dentistry and Pharmaceutical Sciences, Okayama, Japan; ^2^ Princess Margaret Cancer Center, University Health Network, University of Toronto, Toronto, ON, Canada; ^3^ Department of Cell Biology, Okayama University Graduate School of Medicine, Dentistry, and Pharmaceutical Sciences, Okayama, Japan

**Keywords:** ABL—Abelson murine leukemia viral oncogene homolog, Runx2 (runt-related transcription factor 2), tyrosine, phosphorylation, invasion

In the original article, there was a mistake in **Figures 1A, B, D, F**, **2D**, **3B–D, F**, **4D**, **S5A** as published. We noticed that several qPCR data were not correctly normalized since the calculation program of the ΔCt method did not work. The corrected **Figures 1A, B, D, F**, **2D**, **3B–D, F**, **4D**, **S5A** and the equivalent raw data in **Figures S1**–**S4** and **S5A** appears below.

**Figure 1:**
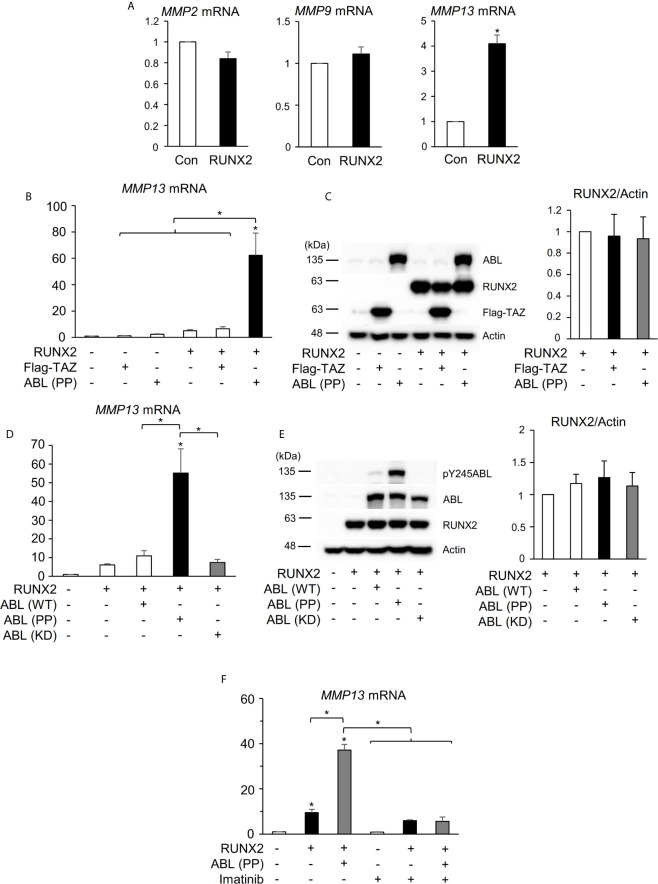
ABL kinase activity is required for RUNX2-mediated MMP13 expression. **(A)** Quantitative PCR analysis of *MMP2, 9, and 13* mRNA expression in HEK293T cells transfected with RUNX2. n = 3. **(B)** Quantitative PCR analysis of *MMP13* mRNA expression in HEK293T cells co-transfected with RUNX2 with or without TAZ or ABL (PP). n = 3. **(C)** HEK293T cells were co-transfected with RUNX2 with or without TAZ or ABL (PP). Whole cell lysates were probed with the indicated antibodies for Western blot analysis. **(D)** Quantitative PCR analysis of *MMP13* mRNA expression in HEK293T cells co-transfected with RUNX2 with or without ABL (WT, PP or KD). n = 3. **(E)** HEK293T cells were co-transfected with RUNX2 with or without ABL (WT, PP or KD). Whole cell lysates were probed with the indicated antibodies for Western blot analysis. **(F)** Quantitative PCR analysis of *MMP13* mRNA expression in HEK293T cells co-transfected with RUNX2 with or without ABL (PP) and cultured in the presence or absence of 10 μM imatinib for 24 hours. n = 3. P values were determined by the unpaired t-test **(A)** or ANOVA with Tukey–Kramer’s *post hoc* test **(B–F)**. Data are presented as means ± SEM. *P < 0.05.

**Figure 2:**
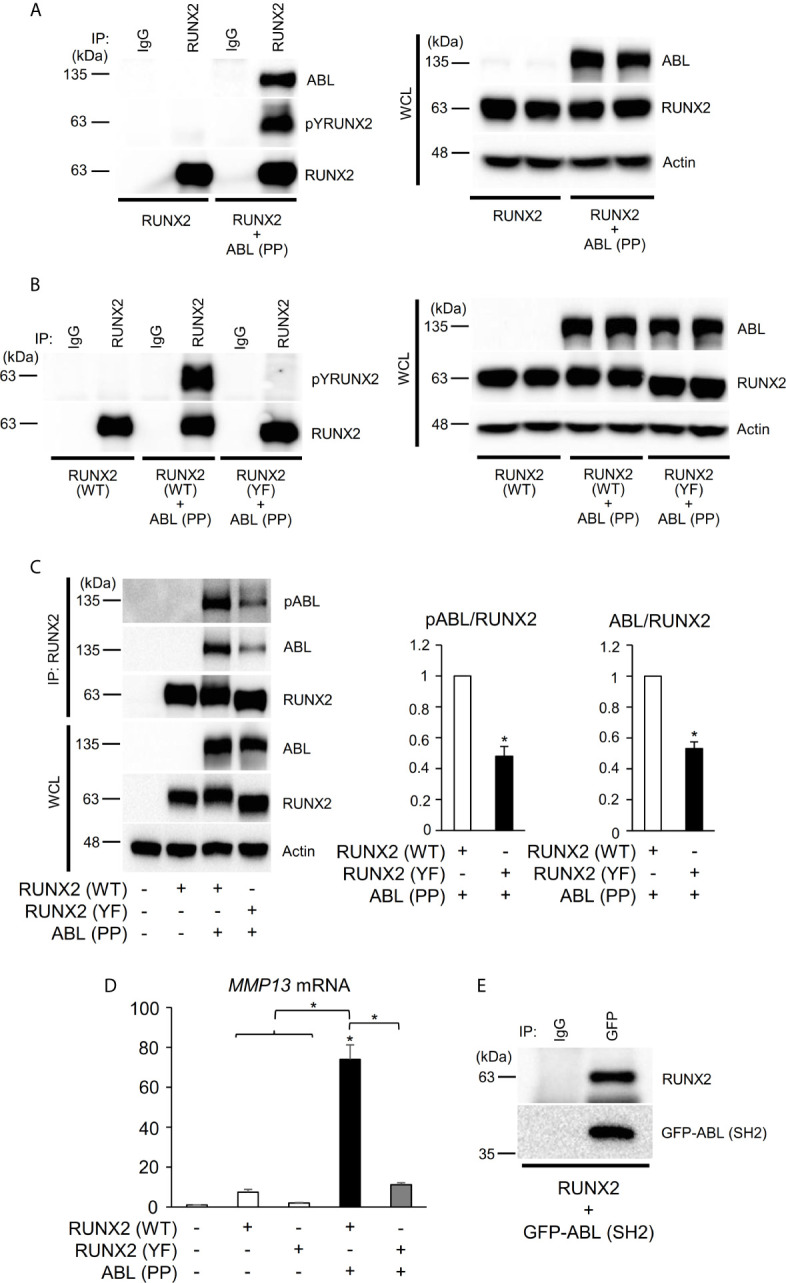
ABL binds to, phosphorylates, and activates RUNX2 through its SH2 domain. **(A–C)** HEK293T cells were co-transfected with wild-type (WT) or all tyrosine to phenylalanine mutant (YF) RUNX2 with or without ABL (PP). RUNX2 immune complexes were probed with an anti-phosphotyrosine (4G10), anti-pY245ABL, anti-ABL or anti-RUNX2 antibody. Whole cell lysates (WCL) were probed with the indicated antibodies for Western blot analysis. **(D)** Quantitative PCR analysis of *MMP13* mRNA expression in HEK293T cells co-transfected with RUNX2 (WT or YF) with or without ABL (PP). n = 3. **(E)** HEK293T cells were co-transfected with RUNX2 with or without GFP-ABL (SH2). GFP-ABL (SH2) immune complexes were probed with an anti-RUNX2 or anti-GFP antibody. P values were determined by ANOVA with Tukey–Kramer’s *post hoc* test. Data are presented as means ± SEM. *P < 0.05.

**Figure 3:**
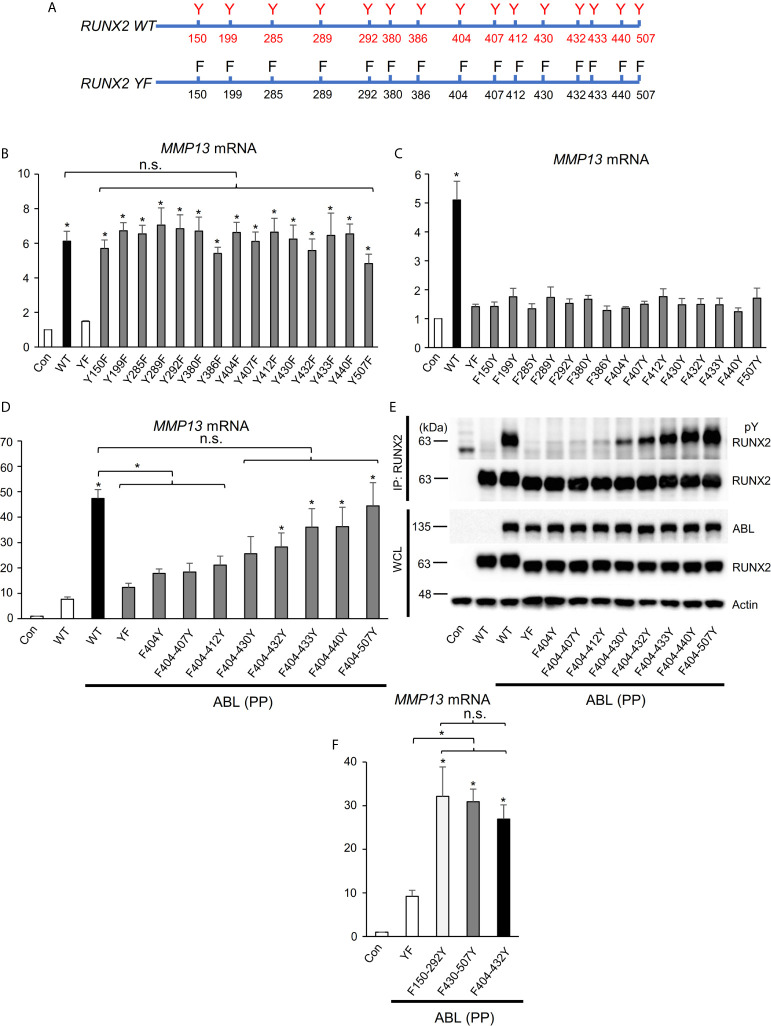
RUNX2 transcriptional activity is dependent on the number of its tyrosine residues phosphorylated by ABL. **(A)** Schematic models of RUNX2 (WT) and RUNX2 (YF). **(B–D, F)** Quantitative PCR analysis of *MMP13* mRNA expression in HEK293T cells co-transfected with the indicated constructs. n = 3. **(E)** HEK293T cells were co-transfected with the indicated constructs and RUNX2 immune complexes were probed with an anti-4G10 or anti-RUNX2 antibody. P values were determined by ANOVA with Tukey–Kramer’s *post hoc* test. Data are presented as means ± SEM. *P < 0.05. ns, no significance.

**Figure 4:**
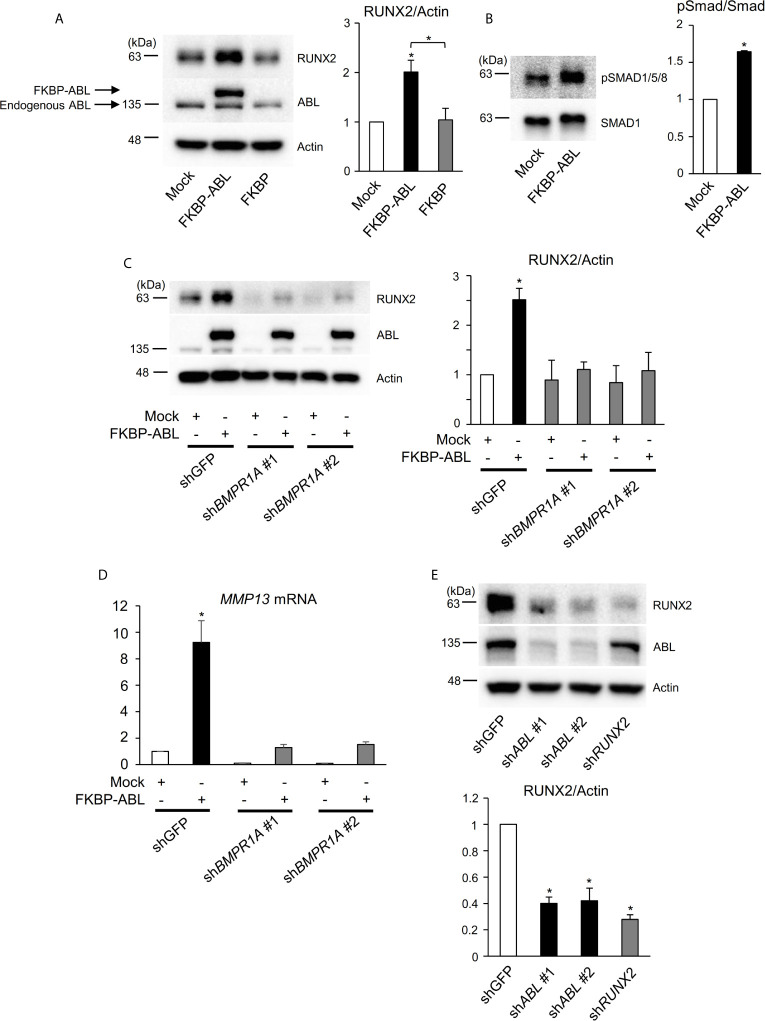
ABL regulates RUNX2 expression through control of the BMP-SMAD pathway. **(A, B)** Saos-2 cells were infected with an empty vector control or an FKBP-ABL- or FKBP-expressing retroviral vector. Whole cell lysates were probed with the indicated antibodies for Western blot analysis. **(C)** Saos-2 cells were infected with an empty vector control or an FKBP-ABL-expressing retroviral vector in the presence of shGFP or sh*BMPR1A*. Whole cell lysates were probed with the indicated antibodies for Western blot analysis. **(D)** Quantitative PCR analysis of *MMP13* mRNA expression in cells in **(C)**. n = 3. **(E)** MDA-MB231 cells were infected with an shGFP-, sh*ABL-* or sh*RUNX2*-expressing vector. Whole cell lysates were probed with the indicated antibodies for Western blot analysis. P values were determined by ANOVA with Tukey–Kramer’s *post hoc* test. Data are presented as means ± SEM. *P < 0.05.

The authors apologize for these errors and state that this does not change the scientific conclusions of the article in any way. The original article has been updated.

## Supplementary Material

The Supplementary Material for this article can be found online at: https://www.frontiersin.org/articles/10.3389/fonc.2021.729192/full#supplementary-material

Supplementary Figure 1 ABL kinase activity is required for RUNX2-mediated MMP13 expression. **(A–D)** Independent raw data of the panels shown in **Figures 1A** **(A)**, **1B** **(B)**, **1D** **(C)** and **1F** **(D)**.

Supplementary Figure 2 ABL binds to, phosphorylates, and activates RUNX2 through its SH2 domain. **(A)** Independent raw data of the panel shown in **Figure 2D**.

Supplementary Figure 3 RUNX2 transcriptional activity is dependent on the number of its tyrosine residues phosphorylated by ABL. **(A–D)** Independent raw data of the panels shown in **Figures 3B** **(A)**, **3C** **(B)**, **3D** **(C)** and **3F** **(D)**.

Supplementary Figure 4 ABL regulates RUNX2 expression through control of the BMP-SMAD pathway. **(A)** Independent raw data of the panel shown in **Figure 4D**.

Supplementary Figure 5 ABL-mediated RUNX2 expression and phosphorylation regulate breast cancer invasion. **(A)** Quantitative PCR analysis of *MMP13* mRNA expression in MDA-MB231 cells infected with an shGFP-, sh*ABL*- or sh*RUNX2*-expressing vector and the independent raw data. n = 3. **(B)** MDA-MB231 cells infected with an shGFP-, sh*ABL*- or sh*RUNX2*-expressing vector were subjected to a Matrigel invasion assay, and invading cells in five independent regions were counted. Representative photographs were taken at 10 × magnification. **(C)** MDA-MB231 cells stably expressing luciferase were infected with an shGFP- or sh*ABL*-expressing vector and injected into the lateral tail veins of BALB/c-nu/nu female mice as described in the methods section. After 4 weeks, the presence of metastases was detected by IVIS, and regions of interest from displayed images were identified and quantified as total photon counts or photons/s. n = 6-7. **(D)** A representative image of H&E staining of the lungs from mice in **(C)**. P values were determined by the unpaired t-test **(C)** or ANOVA with Tukey–Kramer’s post hoc test **(A, B)**. Data are presented as means ± SEM. *P < 0.05.

