# Supplementary material for: RUNX2 Phosphorylation by Tyrosine Kinase ABL Promotes Breast Cancer Invasion
Source: Front Oncol. 2021 May 31;11:665273. doi: 10.3389/fonc.2021.665273 (PMC8201617; doi:10.3389/fonc.2021.665273)
Supplement: Supplementary file 1 [file DataSheet_1.pdf]

Figure S1. ABL kinase activity is required for RUNX2-mediated MMP13 expression. (A-D) Independent raw data of the panels shown in Figure 1A (A), 1B (B), 1D (C) and 1F (D).

Figure S2. ABL binds to, phosphorylates, and activates RUNX2 through its SH2 domain. (A) Independent raw data of the panel shown in Figure 2D.

Figure S3. RUNX2 transcriptional activity is dependent on the number of its tyrosine residues phosphorylated by ABL. (A-D) Independent raw data of the panels shown in Figure 3B (A), 3C (B), 3D (C) and 3F (D).

Figure S4. ABL regulates RUNX2 expression through control of the BMP-SMAD pathway. (A) Independent raw data of the panel shown in Figure 4D.

Figure S5. ABL-mediated RUNX2 expression and phosphorylation regulate breast cancer invasion. (A) Quantitative PCR analysis of *MMP13* mRNA expression in MDA-MB231 cells infected with an shGFP-, sh*ABL*- or sh*RUNX2*-expressing vector and the independent raw data. n = 3. (B) MDA-MB231 cells infected with an shGFP-, sh*ABL*- or sh*RUNX2*-expressing vector were subjected to a Matrigel invasion assay, and invading cells in five independent regions were counted. Representative photographs were taken at 10 × magnification. (C) MDA-MB231 cells stably expressing luciferase were infected with an shGFP- or sh*ABL*-expressing vector and injected into the lateral tail veins of BALB/c-nu/nu female mice as described in the methods section. After 4 weeks, the presence of metastases was detected by IVIS, and regions of interest from displayed images were identified and quantified as total photon counts or photons/s. n = 6-7. (D) A representative image of H&E staining of the lungs from mice in (C). P values were determined by the unpaired t-test (C) or ANOVA with Tukey–Kramer's post hoc test (A,B). Data are presented as means ± SEM. *P < 0.05.

Figure S6. Invasive activity accelerated by ABL is rescued in RUNX2- or MMP13-depleted breast cancer cells. (A-C) Independent raw data of the panels shown in Figure 5A (A), 5B (B) and 5C (C).
