# Supplementary figures and images for: RUNX2 Phosphorylation by Tyrosine Kinase ABL Promotes Breast Cancer Invasion
Source: Front Oncol. 2021 May 31;11:665273. doi: 10.3389/fonc.2021.665273 (PMC8201617; doi:10.3389/fonc.2021.665273)

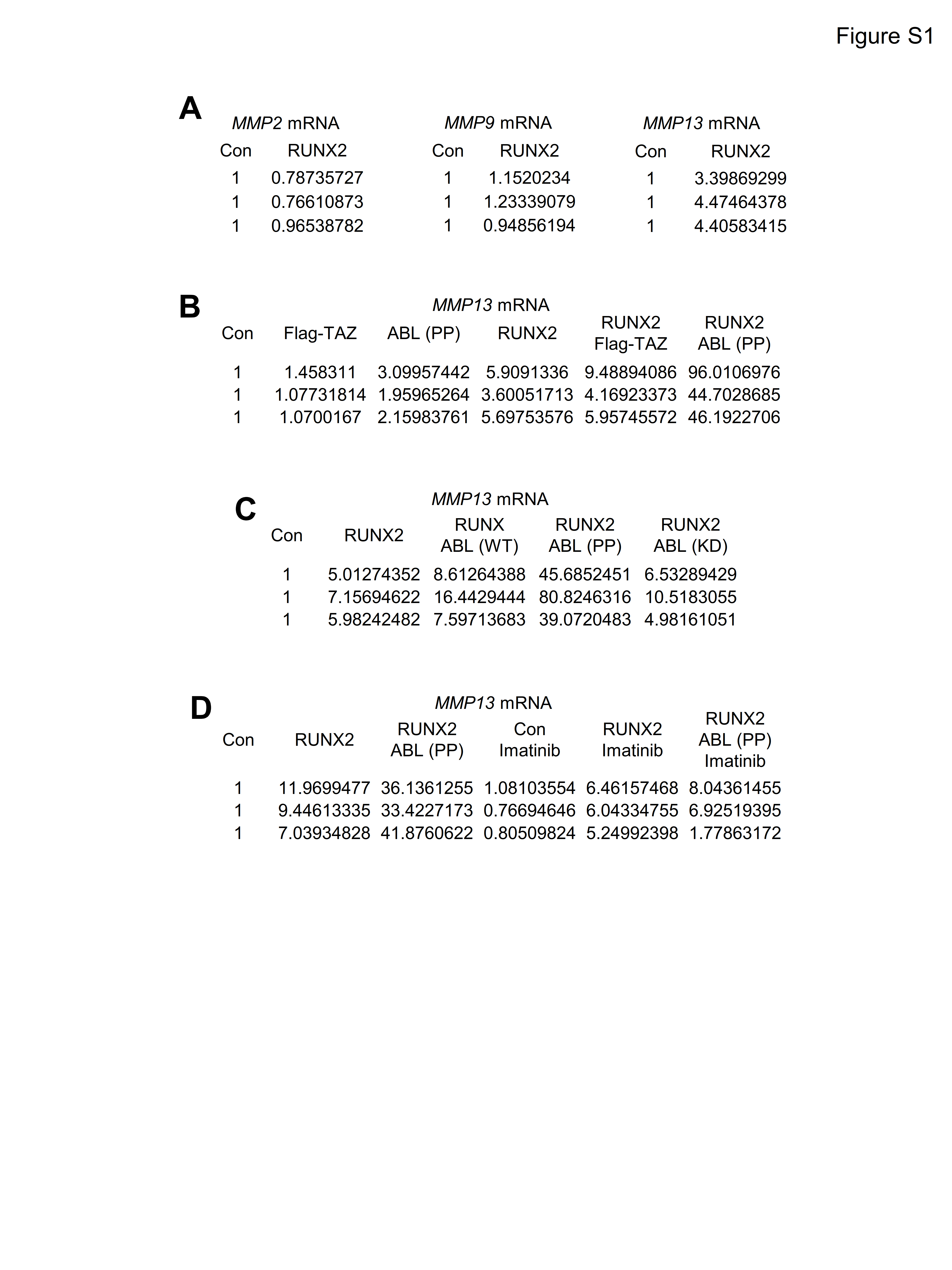

Supplement: Supplementary file 2 [file Image_1.tif]

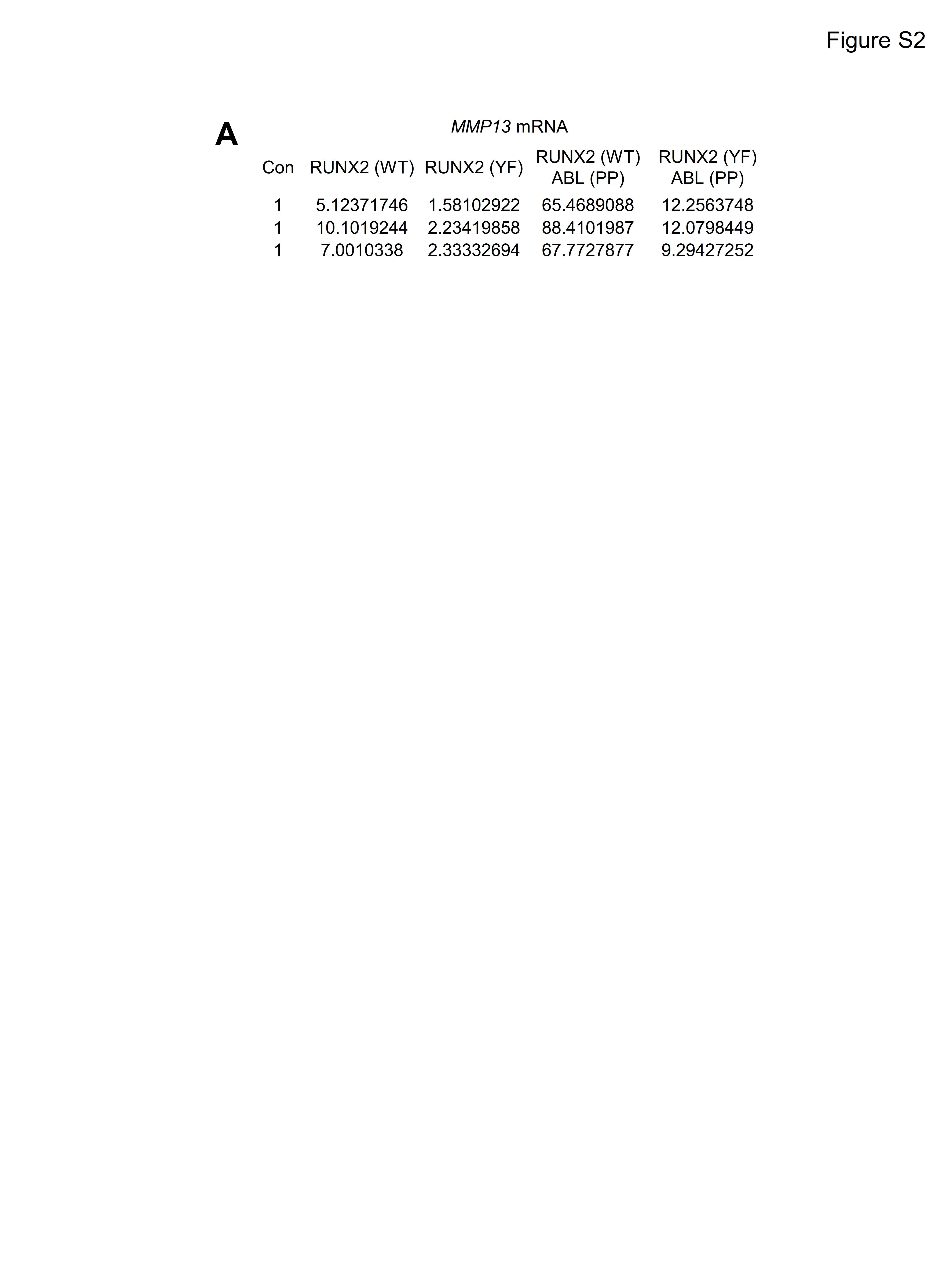

Supplement: Supplementary file 3 [file Image_2.tif]

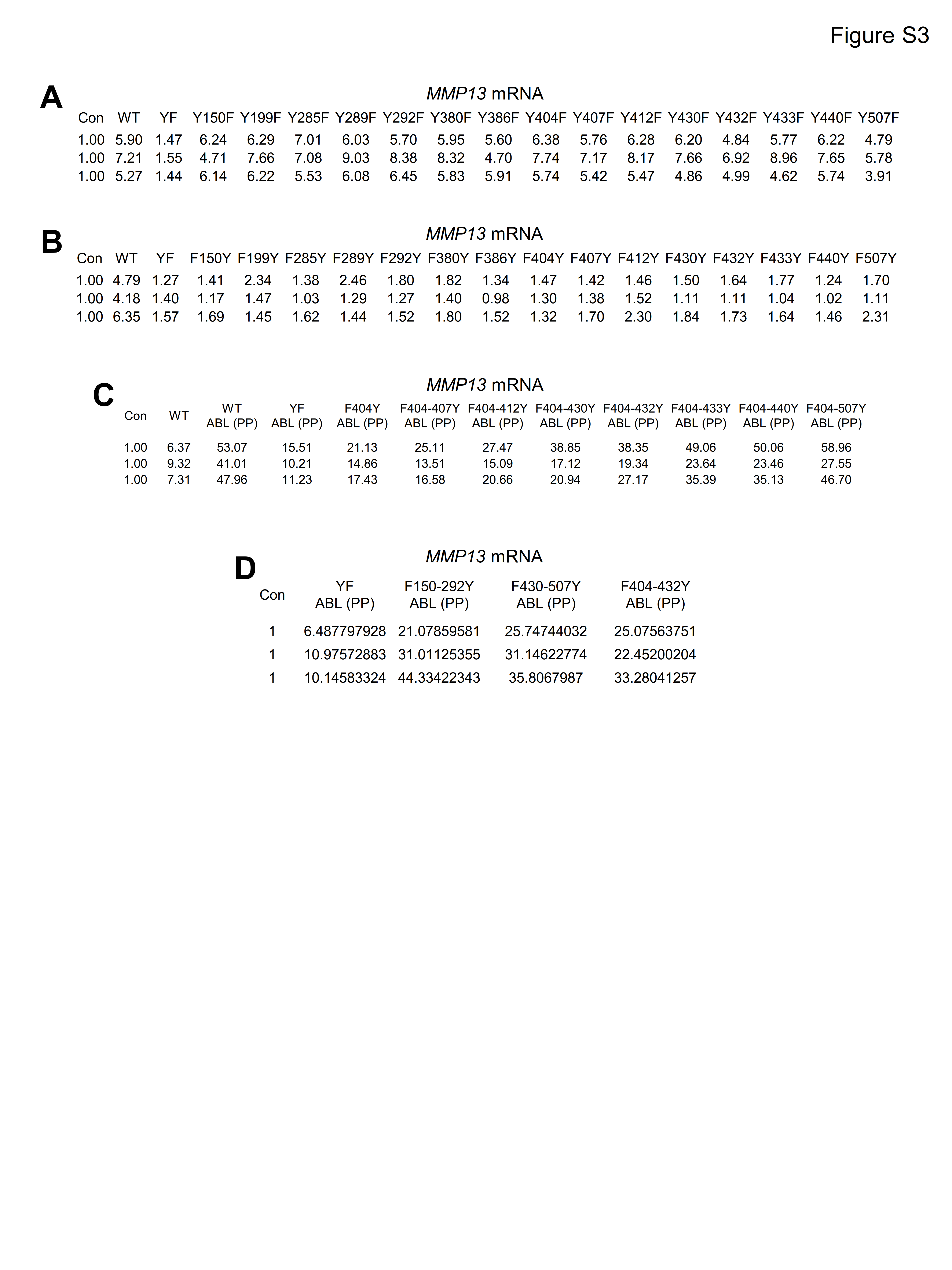

Supplement: Supplementary file 4 [file Image_3.tif]

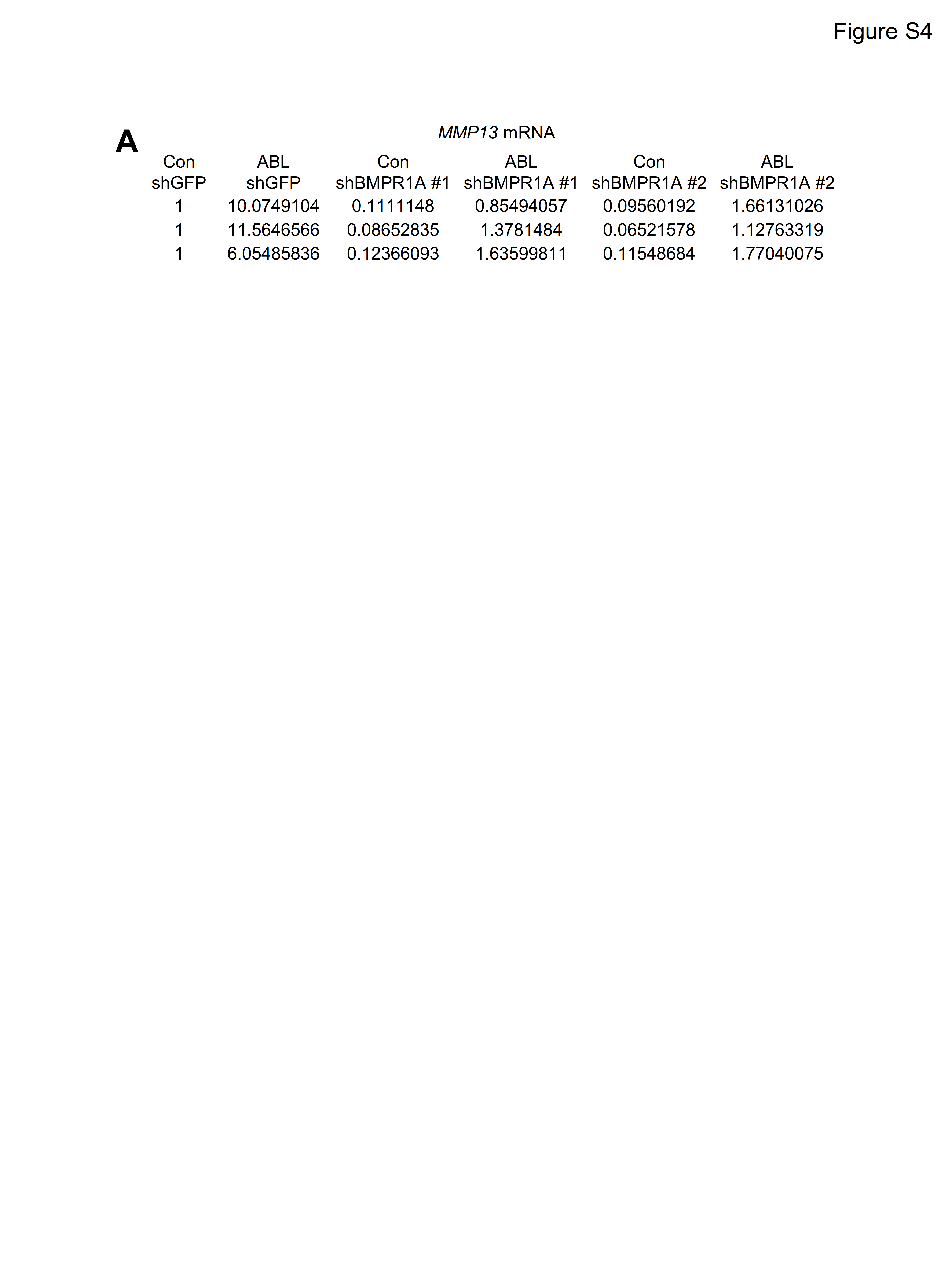

Supplement: Supplementary file 5 [file Image_4.tif]

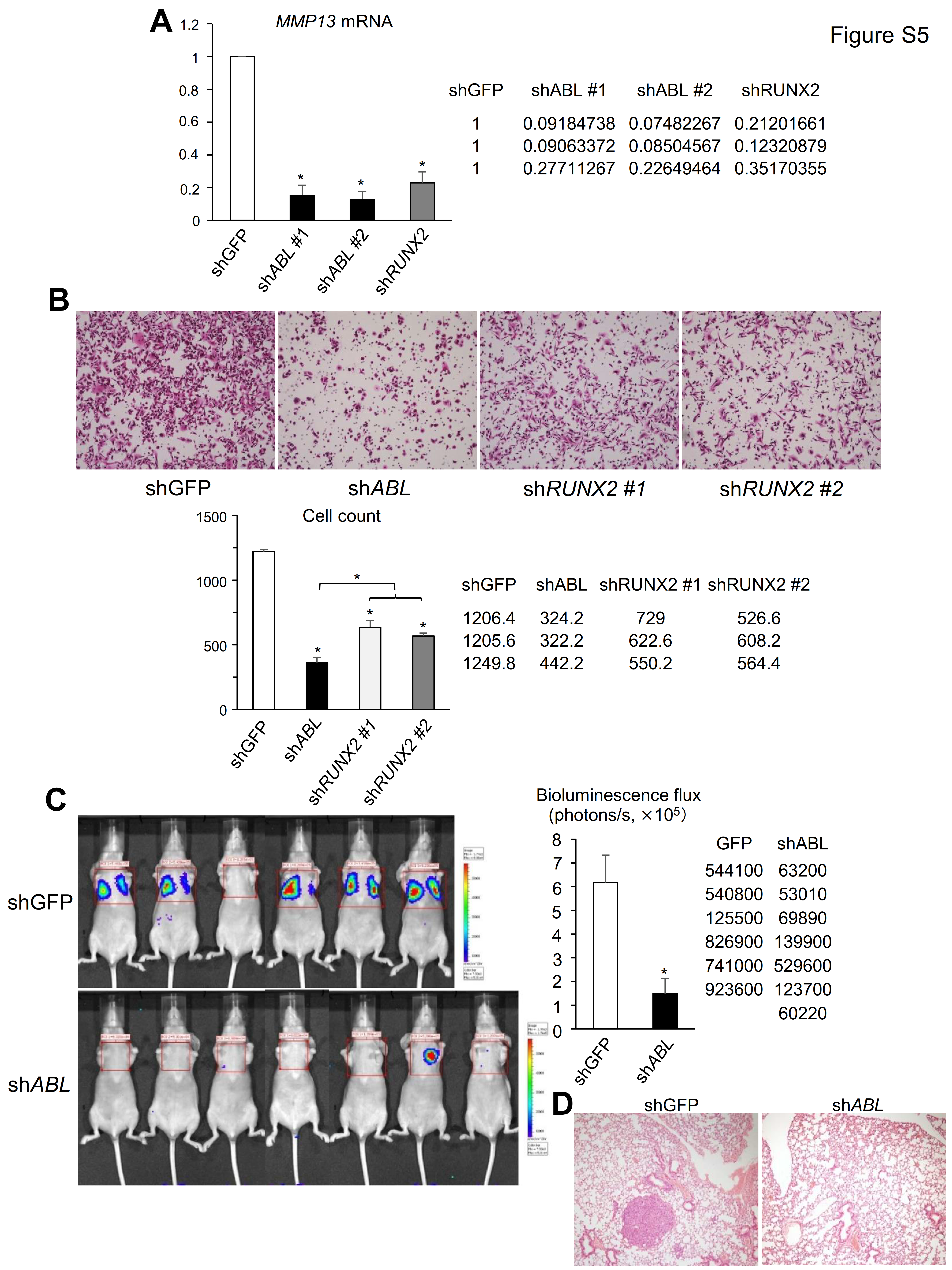

Supplement: Supplementary file 6 [file Image_5.tif]

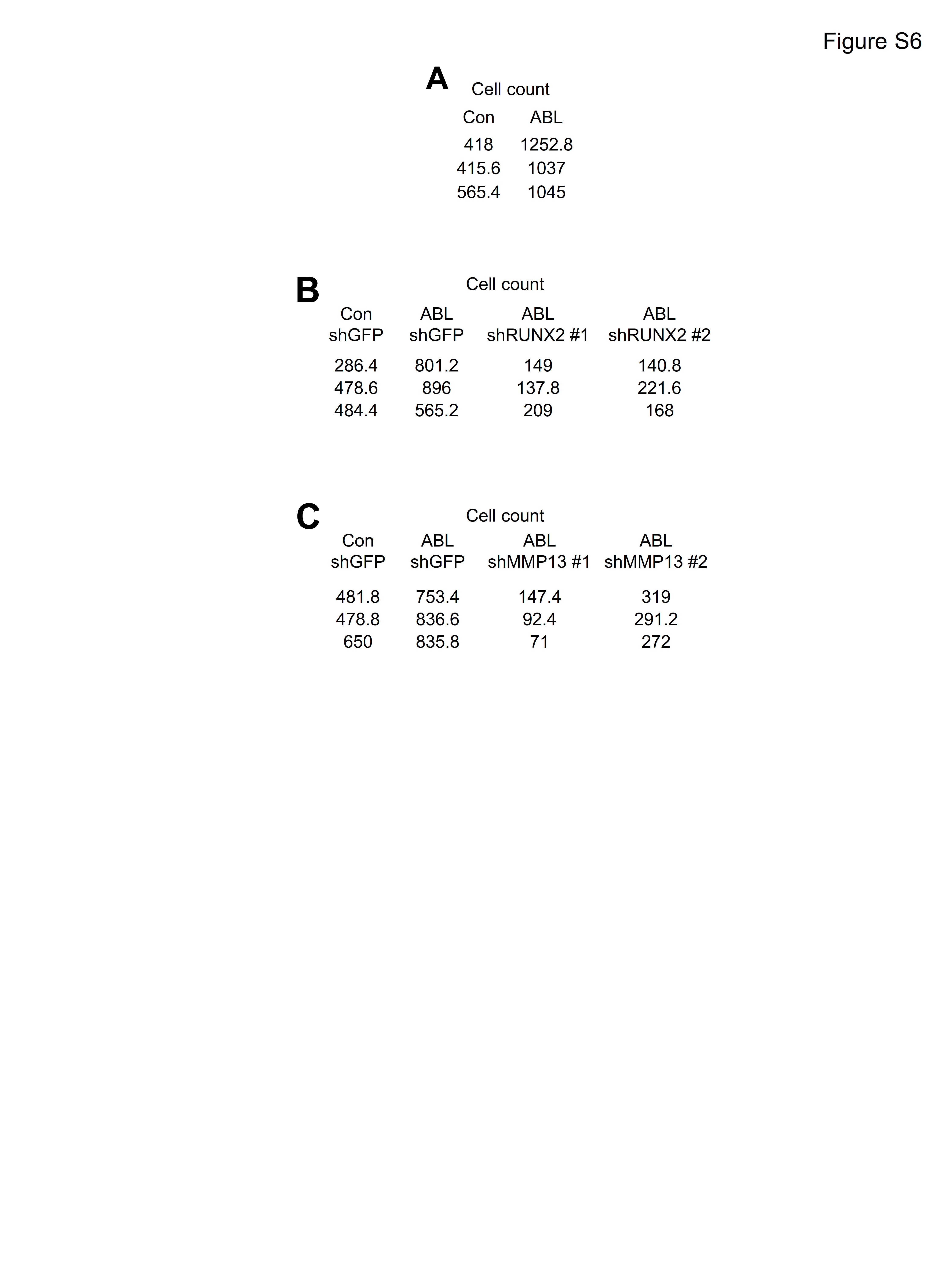

Supplement: Supplementary file 7 [file Image_6.tif]
